# The arrangement of lateral veins along the midvein of leaves is not related to leaf phyllotaxis

**DOI:** 10.1038/s41598-018-34772-2

**Published:** 2018-11-06

**Authors:** Kohei Koyama, Teruhisa Masuda

**Affiliations:** 1 Department of Life Science and Agriculture, Obihiro University of Agriculture and Veterinary Medicine, Inada-cho, Obihiro, Hokkaido 080-8555 Japan; 2 Present Address: Department of Agro-environmental Science, Obihiro University of Agriculture and Veterinary Medicine, Inada-cho, Obihiro, Hokkaido 080-8555 Japan

## Abstract

Positions of leaves along a stem usually adhere to a genetically determined, species-specific pattern known as a leaf phyllotaxis. We investigated whether the arrangement of lateral secondary veins along primary midveins adhered to a species-specific pattern that resembled an alternate or opposite phyllotaxis. We analyzed the venation of temperate dicotyledonous species from different taxonomic groups and chose 18 woody and 12 herbaceous species that have reticulated leaf venation. The arrangement of the lateral veins was neither alternate nor opposite for any of the species. Lateral vein arrangements were instead mixtures of symmetric and asymmetric patterns. Our results show that lateral vein arrangements are related neither to stem-level leaf phyllotaxis (alternate vs. opposite) nor to life form (woody vs. herbaceous). Our results are therefore generally consistent with the canalization hypothesis that the locations of lateral veins are not completely specified genetically prior to leaf formation.

## Introduction

Scaling from an organ to whole-organism function has been one of the main goals of biology^1–7^. In plant scaling theories, modeling plant vascular networks is a fundamental method to scale from plant organs to an individual, because the physiology of vascular networks is linked to all levels of plant physiology, from leaves to trunks^7–14^. Many plant scaling models are based on the simplified assumption of self-similarity of vascular networks^7–9,11,14^. Self-similarity means that branching patterns are similar at multiple scales. It has been suggested that scaling models of stem venation networks can be extended to venation networks in leaves^12^, though deviations from such simple models have been reported^12^. To extend current plant scaling models to leaf vascular networks, it is necessary to quantify the similarity and dissimilarity between two different scales, stem vascular networks and leaf venation networks^12^.

Leaf venation patterns have important physiological implications, because venation patterns are linked with water-, carbohydrate-, and nutrient-transport strategies^15–21^ as well as with the mechanical stability^21–23^ of leaves. Interspecific variations of vein length per unit lamina area (also known as vein density^17,19^), which is one of the determinants of leaf hydraulic conductance^17,18,21,24–26^ and maximum leaf photosynthetic rate^24^, underpin the leaf economics spectrum^16,27–29^. The architecture of reticulated (i.e., net-like) venation is analogous to stem-branching architecture^30^. Because the arrangement of lateral organs (leaves and branches) along the stem is one of the determinants of species-specific branching patterns, it usually follows a species-specific pattern, known as a phyllotaxis, and has also been a subject of studies concerning light interception^31–33^, though several studies have suggested that phyllotaxis per se does not always significantly affect light interception efficiency^32,33^. If leaf venation patterns are species-specific in a way similar to stem-level patterns, modeling approaches that scale functions from veins to trunks could be unified.

However, in contrast to such naive expectations, recent attempts to quantify venation networks^12^ have revealed a discrepancy between simple self-similar models and venation networks. This discrepancy may be explained by the results of recent developmental studies^34,35^ that have identified a likely difference in the mechanisms that underlie the generation of branching nodes of stems and veins. Developmental studies^34,36–38^ have shown that phyllotaxis or the regular arrangement of leaves (and lateral branches) along a stem is a consequence of self-organized spacing between auxin concentration peaks, each of which generates a single leaf primordium at the apex. The results of those studies have shown that during the generation of phyllotaxis, each lateral organ becomes spontaneously separated from nearby lateral organs around the growing apical meristem. Canalization models of venation networks^34,39–45^ have suggested that gullies or canals are analogous to veins, which are polarized by auxin flow (i.e., “with-the-flux polarization”^40,46^). Recently, canalization models of venation have been further unified with phyllotaxis models of auxin flow. In those unified models (i.e., “dual polarization” models^15,34,35,46^), the spacing of leaves and formation of midveins occur concurrently^34,46^. It is, however, still unclear whether lateral venation patterns exhibit consistent morphologies that correspond to phyllotaxis.

Based on our review of previous studies, we suggest that there is an inherent difference between the mechanisms that generate branching points on the main axes of stems and midveins. In the case of stem branching or generation of phyllotaxis, the branching nodes are generated in the region near the growing apexes, as described above. In contrast to stem branching, in vein formation, the generation of branching nodes along the midvein, to which lateral secondary veins are connected, is not confined to the growing apex or the edge of a leaf. Instead, branching nodes on the midvein are formed by the coalescence of flows from the two opposite sides of a leaf lamina into the midvein^15,35,45^. Those studies have therefore indicated that the branching points on the midvein, from which lateral veins bifurcate, may not be controlled by the same spacing mechanism responsible for phyllotaxis. Indeed, it has long been recognized that unlike phyllotaxis, which is a genetically determined species-specific trait, the arrangement of lateral veins may not be strictly determined genetically^35^ but instead is subject to unpredictable noise during development^40^. Despite the fact that both phyllotaxis and venation appear to be controlled by auxin flows^34,46^, the mechanisms responsible for branching of the main axis may therefore differ.

In this study, to quantify the similarity or dissimilarity between stem-level phyllotaxis and leaf-level venation patterns, we investigated the arrangement of lateral veins along the midvein. Specifically, we asked the following questions: (1) does the arrangement of secondary veins reflect a species-specific, genetically determined pattern that is analogous to either an alternate or opposite phyllotaxis? (2) Is the venation pattern consistent with stem-level leaf phyllotaxis?

## Methods

### Species and sampling site

In 2017 and in 2018, we investigated 18 woody and 12 herbaceous species that had clearly visible reticulate venation: 18 had an alternate and 12 had an opposite phyllotaxis (Table 1). Hereafter, the names of the species are abbreviated by their genus names. Each species was from a different family, except for two species (*Fraxinus* and *Ligustrum*) that both belonged to the family Oleaceae. Among the woody species, three (*Aucuba*, *Lonicera*, and *Rhododendron*) were evergreens, and the rest were deciduous species.

**Table 1:** List of the species.

Code	Species	Family	Life form				
Ame	*Amphicarpaea bracteata (*L.) Fernald subsp. *edgeworthii* (Benth.) H.Ohashi var. *japonica* (Oliv.) H.Ohashi	Fabaceae	H(herbaceous), L(liana)				
Atg	*Acer ginnala* Maxim. var. *aidzuense* (Franch.) Pax	Sapindaceae	W(woody), D(deciduous)				
Auc	*Aucuba japonica* Thunb.	Aucubaceae	W,E(evergreen)				
Bid	*Bidens* sp.^a^	Asteraceae	H				
Bpt	*Betula platyphylla* Sukaczev var. *japonica* (Miq.) H.Hara	Betulaceae	W, D				
Cay	*Cayratia japonica* (Thunb.) Gagnep.	Vitaceae	H, L				
Ces	*Cerasus sargentii* (Rehder) H.Ohba	Rosaceae	W, D				
Coc	*Cornus controversa* Hemsl. ex Prain	Cornaceae	W, D				
Cpa	*Chenopodium album* L.	Amaranthaceae	H				
Cyr	*Cynanchum rostellatum* (Turcz.) Liede & Khanum	Apocynaceae	H, L				
Elc	*Eleutherococcus senticosus* Maxim.	Araliaceae	W, D				
Eua	*Euonymus alatus* (Thunb.) Siebold	Celastraceae	W, D				
Fas	*Fallopia sachalinensis* (F.Schmidt) Ronse Decr.	Polygonaceae	H				
Fms	*Fraxinus mandshurica* Rupr.	Oleaceae	W, D				
Hyp	*Hydrangea paniculata* Siebold	Hydrangeaceae	W, D				
Jms	*Juglans mandshurica* Maxim. var. *sachalinensis* (Komatsu) Kitam.	Juglandaceae	W, D				
Jpc	*Justicia procumbens* L.	Acanthaceae	H				
Lgo	*Ligustrum obtusifolium* Siebold & Zucc.	Oleaceae	W, D				
Lon	*Lonicera japonica* Thunb.	Caprifoliaceae	W, E, L				
Mkb	*Magnolia kobus* DC. var. *borealis* Sarg.	Magnoliaceae	W, D				
Oeb	*Oenothera biennis* L.	Onagraceae	H				
Pas	*Paederia foetida* L.	Rubiaceae	H, L				
Piq	*Picrasma quassioides* (D.Don) Benn.	Simaroubaceae	W, D				
Pps	*Populus suaveolens* Fisch.	Salicaceae	W, D				
Qmc	*Quercus crispula* Blume	Fagaceae	W, D				
Rhd	*Rhododendron brachycarpum* D.Don ex G.Don	Ericaceae	W, E				
Scs	*Scutellaria strigillosa* Hemsl.^b^	Lamiaceae	H				
Sol	*Solanum* sp.^c^	Solanaceae	H				
Sym	*Symphytum officinale* L.^d^	Boraginaceae	H				
Udj	*Ulmus davidiana* var. *japonica* (Rehder) Nakai	Ulmaceae	W, D				
**Code**	**Phyllotaxis**	**Leaf**	**Types of venation** ^e^	**Height (m)**	**Site** ^f^	***L*** ^g^	***N*** ^h^
Ame	alternate	ternate	basal actinodromous	1.1–1.9	1, 2	60	454
Atg	opposite	simple	basal actinodromous^i^	2.9–5.0	1	15	173
Auc	opposite	simple	semicraspedodromous	1.9–2.2	7	15	223
Bid	opposite	pinnate	craspedodromous	0.5–1.2	1	15	321
Bpt	alternate	simple	craspedodromous	9.1–13.3	2	15	199
Cay	alternate	palmate	craspedodromous	0.3–1.0	7	15	179
Ces	alternate	simple	semicraspedodromous	1.7–9.3	1	15	223
Coc	alternate	simple	eucamptodromous	3.4–6.4	1	15	191
Cpa	alternate	simple	suprabasal actinodromous	1.0–2.3	2	50	234
Cyr	opposite	simple	semicraspedodromous^i^	0.5–1.1	2, 3	15	225
Elc	alternate	palmate	semicraspedodromous	1.7–2.5	3	15	195
Eua	opposite	simple	semicraspedodromous	1.0–1.7	1	15	161
Fas	alternate	simple	semicraspedodromous	2.7–2.9	3	15	306
Fms	opposite	pinnate	semicraspedodromous	5.0–27	2	15	344
Hyp	opposite	simple	eucamptodromous	1.9–3.1	5	15	155
Jms	alternate	pinnate	craspedodromous	1.7–15	1, 2	15	522
Jpc	opposite	simple	eucamptodromous	0.4–0.6	7	50	414
Lgo	opposite	simple	brochidodromous	1.4–3.4	1	15	180
Lon	opposite	simple	semicraspedodromous	0.5–2.4	7	50	418
Mkb	alternate	simple	craspedodromous	2.8–9.6	3, 5	15	250
Oeb	alternate	simple	semicraspedodromous	1.3–1.8	2	15	354
Pas	opposite	simple	semicraspedodromous	n.a.^j^	7	50	464
**Code**	**Phyllotaxis**	**Leaf**	**Types of venation** ^e^	**Height (m)**	**Site** ^f^	***L*** ^g^	***N*** ^h^
Piq	alternate	pinnate	semicraspedodromous	2–6	7	15	219
Pps	alternate	simple	craspedodromous	13–15	2, 3	15	161
Qmc	alternate	simple	craspedodromous	2.4–6.4	1	15	413
Rhd	alternate	simple	craspedodromous	2–3	1, 3	15	350
Scs	opposite	simple	semicraspedodromous	0.2–0.5	1	50	324
Sol	alternate	simple	semicraspedodromous	0.2–0.5	4	50	347
Sym	alternate	simple	reticulodromous	0.5–0.7	6	15	189
Udj	alternate	simple	craspedodromous	13–15	2	15	418

^a^We tentatively identified this species as *Bidens frondosa*, which has recently been reported as being distributed in Obihiro city^67^. However, because we were unsure of the identification of this species, we identified it only to the genus level.

^b^We did not distinguish *Scutellaria strigillosa* from *S. strigillosa* var. *yezoensis* (synonym: *S. yezoensis*).

^c^We tentatively identified this species as *Solanum nigrum*, but because there are several species within the same genus that are difficult to distinguish from each other^68^ we identified it only to the genus level.

^d^We did not distinguish *Symphytum officinale* from a hybrid of *S*. *officinale* and *S*. *asperum*, which may also be found in Japan^69^.

^e^Following Leaf Architecture Working Group (LAWG)^30^.

^f^Sampling sites (1. The Forest of Obihiro. 2. The university. 3. The Urikari River. 4. Roadside near Obihiro station. 5. Tokachi Ecology Park. 6. The Kikankono River. 7. The Taguri River).

^g^*L*: total number of leaves or leaflets analyzed for each species.

^h^*N*: total number of lateral veins analyzed for each species.

^i^These two species (*Acer* and *Cynanchum*) had several thick veins that originated radially from the base of the leaf lamina and terminated at the tip of the lobes. Those may not have been basal primaries, if the strict definition by LAWG^30^ is followed, because the thickest one usually accounted for less than 74% of the width of the midvein. However, because venations of maples with lobed leaves have often been classified as actinodromous, the venations of these two species were also classified as actinodromous.

^j^Not available: multiple vines wound together.

The plants were sampled from seven locations in Japan: (1) the Forest of Obihiro (Obihironomori), a plantation forest that included a mixture of planted and regenerated trees. The sampling locations included the roadsides inside and around the forest; (2) the campus of the Obihiro University of Agriculture and Veterinary Medicine, including an experimental garden at the Field Center of Animal Science and Agriculture; (3) a roadside near the Obihiro station; (4) the riparian zone of the Urikari River; (5) Tokachi Ecology Park, which is located in the riparian zone of the Tokachi River; (6) the riparian zone of the Kikankono River; and (7) a roadside near the Taguri River.

The sites (1)–(6) were located in Obihiro City or in Otofuke Town in Hokkaido in a cool-temperate region in Japan. These six sites were within 10 km from the Japan Meteorological Agency Obihiro Weather Station (42°52′N 143°10′E, altitude: 76 m a.s.l.). The mean annual temperature and precipitation at the weather station during 1998–2017 were 7.2 °C and 937 mm, respectively. The site (7) was located in Sakura City in Chiba prefecture in a warm-temperate region in Japan. This site was ca. 4 km from the Japan Meteorological Agency AMeDAS Sakura Observatory (N35°44′ E140°13′, 5 m a.s.l.). The mean annual temperature and precipitation at the observatory during 1999–2017 were 14.9 °C and 1469 mm, respectively. The weather data were taken from the Japan Meteorological Agency homepage, viewed 2 March 2018.

### Samplings and measurements

For each species, 15 leaves (three individuals and five leaves or leaflets per individual) were measured, except for seven species (*Amphicarpaea*, *Chenopodium*, *Justicia*, *Lonicera*, *Paederia*, *Scutellaria*, and *Solanum*) that had relatively few lateral veins on each lamina. For each of those seven species, 50 or 60 leaves (five individuals and 10 or 12 leaves or leaflets per individual) were measured to ensure that sufficient numbers of lateral veins were measured (Table 1). For two clonal species, *Cynanchum* and *Paederia*, each individual ramet was sampled from separate places to ensure that it was sampled from a different genet. For other clonal species, *Cayratia* and *Scutellaria*, the ramets were sampled from two places for each species because of time constraints. For the other clonal species, *Fallopia*, each ramet was sampled from different stands that were located in the same riparian zone of the Urikari River. For *Cayratia*, *Scutellaria*, and *Fallopia*, the total numbers of genets were therefore unknown.

Living shoots of trees, or for herbaceous species usually entire ramets, were sampled at the study site with pruning scissors or a long-reach pruner. Immediately after sampling, shoots or ramets were stored in closed plastic bags with wet paper towels to prevent desiccation and then transported to our laboratory or to a dormitory. Leaves were scanned as color images with A4 flatbed scanners (CanoScan LiDE 210 or LiDE 220, Canon, Tokyo) at a resolution of 400 dpi. The scanning was usually conducted on the same day as the sampling. Because of time constraints, in some cases samples in closed plastic bags were stored in a refrigerator, and the leaves were scanned within two days of the sampling day.

### Definition of vein orders

For simple leaves, we followed the definition of primary and secondary veins articulated by the Leaf Architecture Working Group^30^, which is an updated version of the standard definition by Hickey^47^, but with a few modifications. (1) For each leaf, the midvein at the center of each lamina was recognized as a primary vein. (2) For leaves or leaflets that had actinodromous venation (*Acer*, *Amphicarpaea*, and *Chenopodium*), for which more than one primary vein originated at the base of the leaf lamina, other basal (or nearly basal) veins were also recognized as primaries. Similarly, for *Cynanchum*, thick basal lateral veins were not recognized as lateral veins. (3) Tertiary veins were defined as the widest veins that filled the field of the leaf lamina, and secondary veins were defined as veins that were at intermediate positions between the primary and tertiary veins. In this study, we defined “lateral veins” as secondary veins that originated from the midvein and ran toward the leaf margin. For leaves that had more than one primary vein, only the midveins were measured. Intersecondary veins^30^ that were considerably thinner than other lateral veins and did not reach the leaf margin were not considered to be lateral veins in the present analysis. If a single lateral vein bifurcated into two or more lateral veins on the way toward the leaf margin, it was still considered as a single lateral vein, and the point from which that lateral vein originated from the midvein (central vein) was measured.

For compound leaves, following the model by Runions *et al*.^48^, we regarded each leaflet as equivalent to a single leaf for the purposes of this study. Nonetheless, because a lateral vein of a leaflet may not be equivalent to the lateral vein of a simple leaf, we performed the following preliminary analysis to determine whether we could regard a leaflet as equivalent to a leaf, at least for purposes of this study. We used leaflets of *Amphicarpaea* (hog-peanut), which had ternate leaves, and we separately analyzed the terminal leaflets and compared them to the leaflets from all positions (including the terminal, the left-positioned, and the right-positioned leaflets; we followed the left-right terminology by Martinez *et al*.^49^). We considered the lateral veins of a terminal leaflet to be equivalent to those of a simple leaf. As shown in the Results section, we obtained similar results when we analyzed only the terminal leaflets and when we analyzed leaflets from all positions. The same procedure described above was therefore performed to determine the midvein and the lateral veins for each leaflet. For a compound leaf, we therefore defined the “midvein” to be the vein at the center of each leaflet.

### Image analysis

The digital images were analyzed by using ImageJ ver 1.51n^50^, and statistical analyses were performed with R ver 3.5.1^51^ For each leaf, the image of either the adaxial or abaxial surface was measured, depending on the distinguishability of the lateral veins. We manually determined the coordinates of all the points at which lateral veins bifurcated from the midvein by using the “point tool” of ImageJ. For each of those points, two sets of distances (*d*_opposite_ and *d*_same_) were calculated from the coordinates. For each vein, the ratio (*r*) between those two distances was calculated as follows:

1 $$r=\frac{d{\rm{opposite}}}{d{\rm{same}}}$$

**Figure 1:**
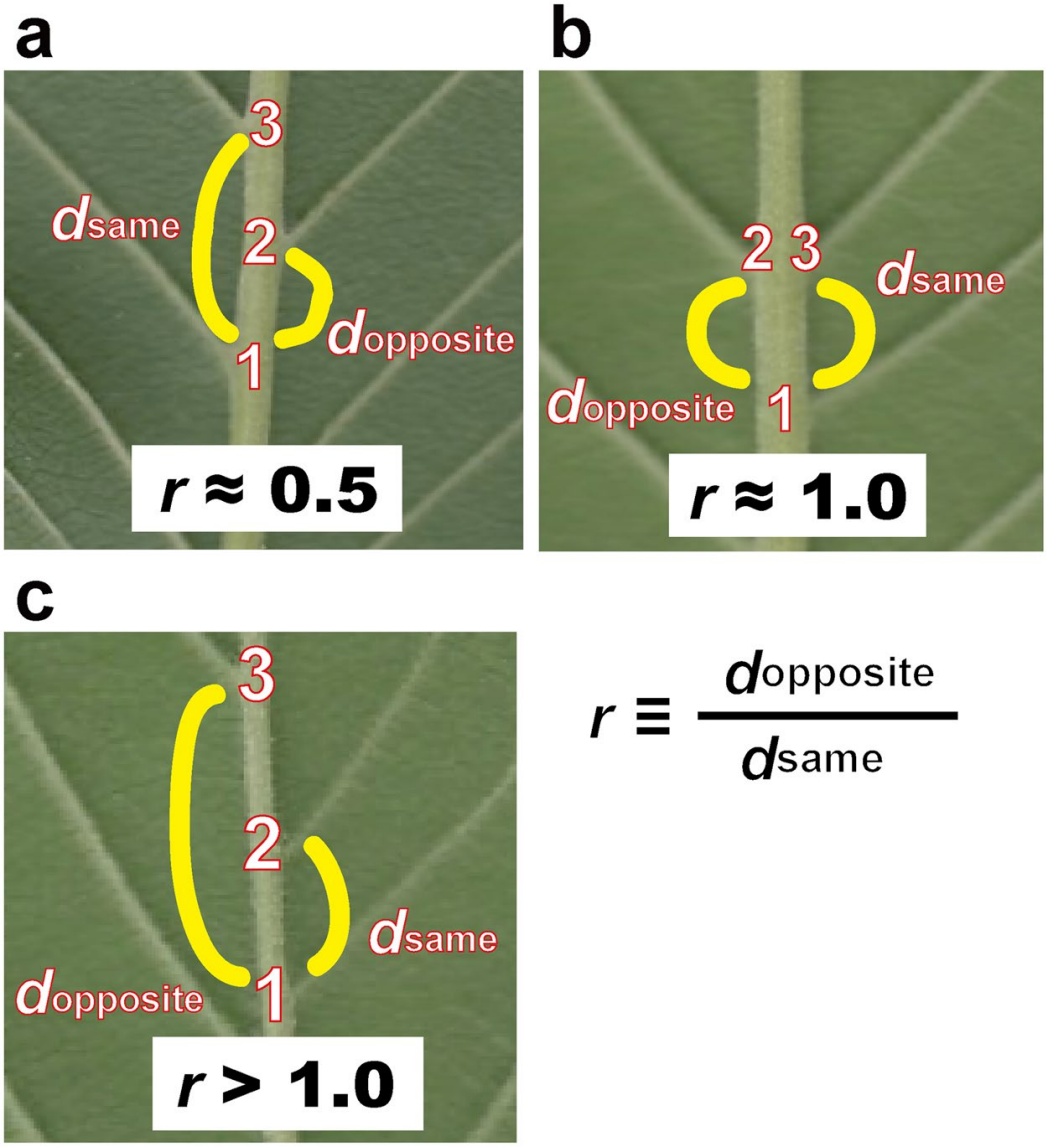
Arrangement of lateral secondary veins along the midvein. Leaves of *Ulmus* are shown as examples. In the panel (a), *d*_opposite_ is the distance between the first lateral vein (1) and the next lateral vein (2) on the opposite side of the central vein, and *d*_same_ is the distance between the first lateral vein (1) and the next lateral vein (3) on the same side of the central vein. For each lateral vein, the ratio (*r*) is calculated as *d*_opposite_ divided by *d*_same_. (**a**) *r* ≈ 0.5 indicates that a lateral vein on one side of a leaf lamina originated from approximately halfway between the two points of origin of the lateral veins on the opposite side. (**b**) *r* ≈ 0 or *r* ≈ 1 indicates that a lateral vein on one side of a leaf lamina originated at approximately the same point as the other lateral vein on the opposite side. (**c**) We also observed a few cases in which two successive lateral veins were on the same side of a leaf lamina. This irregular pattern is expressed as *r* > 1. (Photographs by Kohei Koyama).

If the lateral veins were arranged in the same way as the alternate phyllotaxis, the distribution of *r* should be unimodal, with a single mode at an *r* of 0.5 (Fig. 1a). In contrast, if the lateral veins were arranged in the same way as the opposite phyllotaxis, then the expected distribution of *r* would be bimodal, with two modes at *r*’s of 0 and 1 (Fig. 1b). As shown in the Results, the shapes of the *r* distributions among the species investigated varied continuously between those two extremes. For each species, we therefore determined the degree of departure from unimodality as an index of departure from the alternate-like vein arrangement. To evaluate unimodality, all the values of *r* were pooled for each species, and the distribution was examined with a violin plot (by using the R package ‘vioplot’^52^), which is useful for visualizing a difference between unimodal and bimodal distributions^53^, combined with a bee swarm plot^54^ (by using the R package ‘beeswarm’^54^), which is useful for visualizing an entire dataset without any information loss. The analysis was carried out with Hartigan’s dip test of unimodality^55^ (by using the R package ‘diptest’^56^). We followed the argument by Freeman and Dale^57^ that the *p*-value of the dip test can be used as a measure of departure from unimodality; a small *p*-value indicates a deviation from a unimodal distribution, and in the present context, was considered to indicate a departure from an alternate-like vein arrangement. We therefore tested whether the *p*-values differed between species with different stem-level phyllotaxes (alternate vs. opposite) or between woody and herbaceous species with a two-sided nonparametric Brunner–Munzel test^58^ by using the R package ‘lawstat’^59^.

We occasionally observed cases in which two successive lateral veins were on the same side of a leaf lamina (Fig. 1c). This irregular pattern is expressed as *r* > 1. We hypothesized that if those irregular cases were generated by a systematic rule, they should appear at approximately the same positions on each pair of left- and right-positioned leaflets of ternately compound leaves of *Amphicarpaea* (i.e., they should appear as mirror images). We therefore compared the relative positions (defined as the distance between the position and the leaflet base divided by the length of the leaflet, 0 = base and 1 = tip) at which this *r* > 1 pattern appeared for each case to determine whether the *r* > 1 cases appeared as mirror images on the compound leaves.

## Results

**Figure 2:**
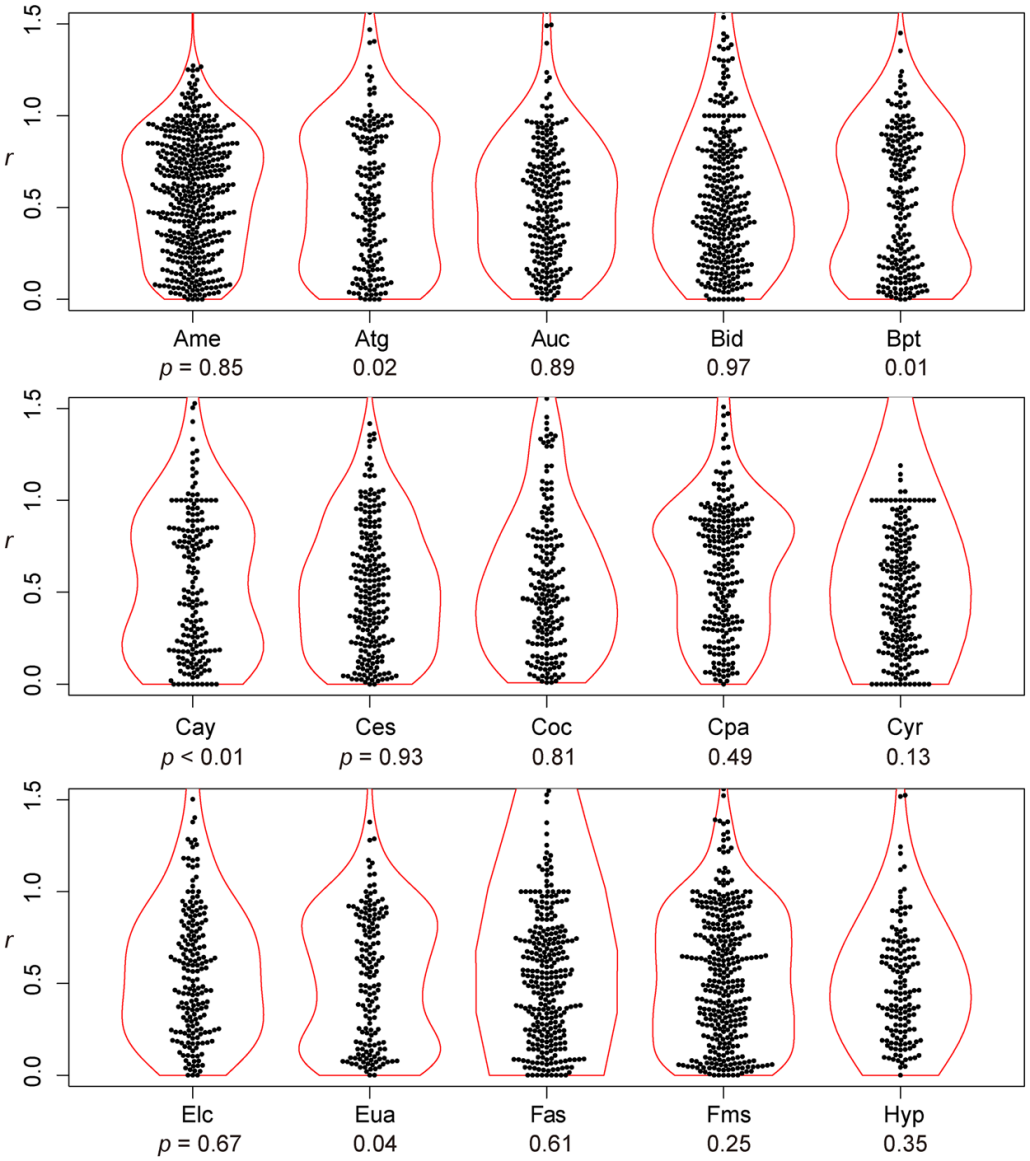
Distributions of the relative position of lateral veins (*r*). The values for *r* ≤ 1.5 are shown. The codes of species names are shown in Table 1. For each species, each value of *r* for a lateral vein is shown as a filled circle, which is arranged so as not to overlap others (i.e., a bee swarm plot), and the violin-shaped pair of red curves shows the estimated probability density distribution (i.e., violin plot); the wider the violin at a particular height, the more common the *r* values are around that value. The *p*-values for the Hartigan’s dip test of unimodality are shown under the code of each species; lower *p*-values indicate higher departure from unimodality. Species are arranged in alphabetical order, and data for the first fifteen species (Ame–Hyp) are shown (see Fig. 3 for Jms–Udj).

**Figure 3:**
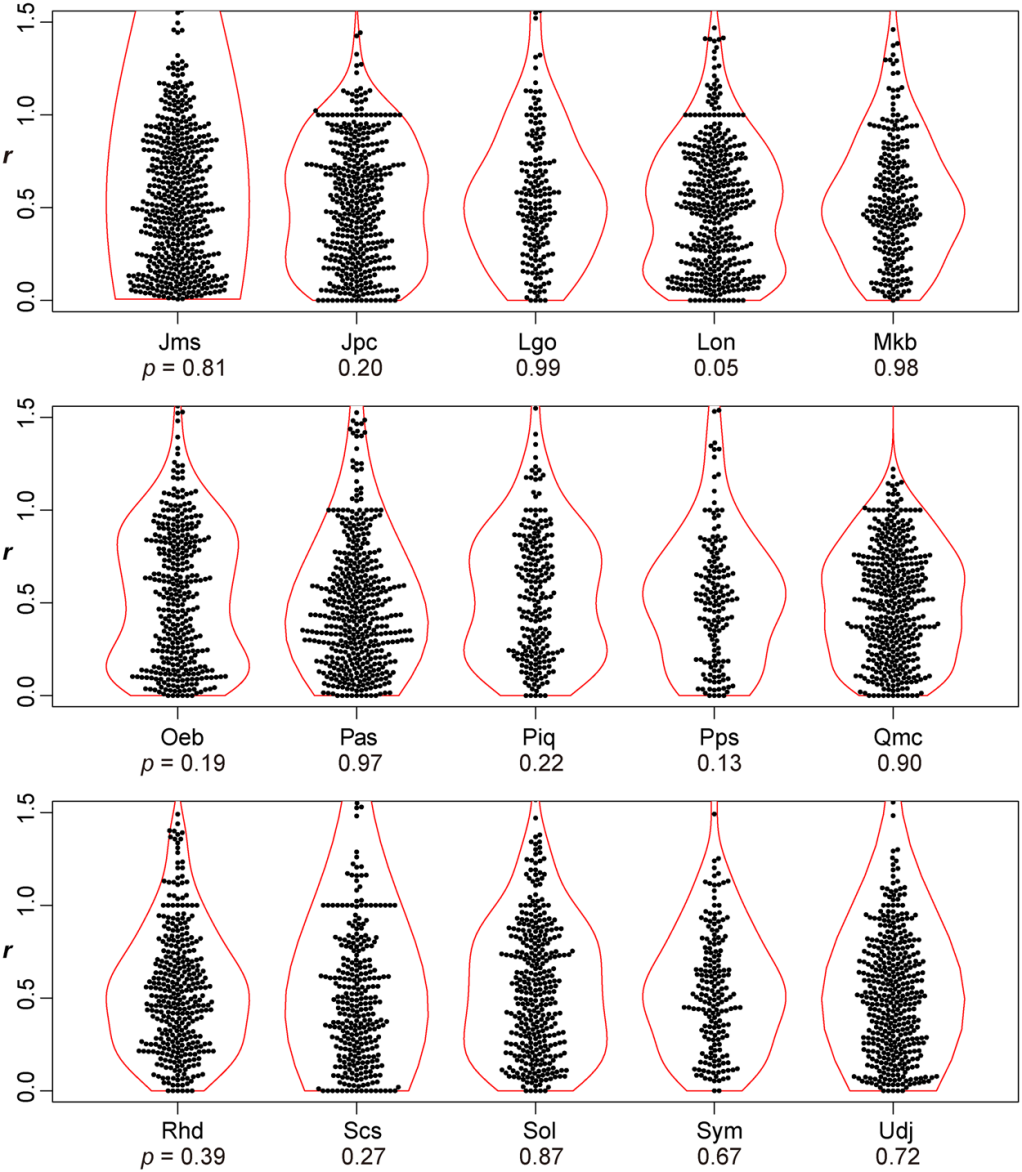
Distributions of the relative position of lateral veins (*r*) (see Fig. 2 for legend). The values for *r* ≤ 1.5 are shown. Data for the last fifteen species (Jms–Udj) are shown.

**Figure 4:**
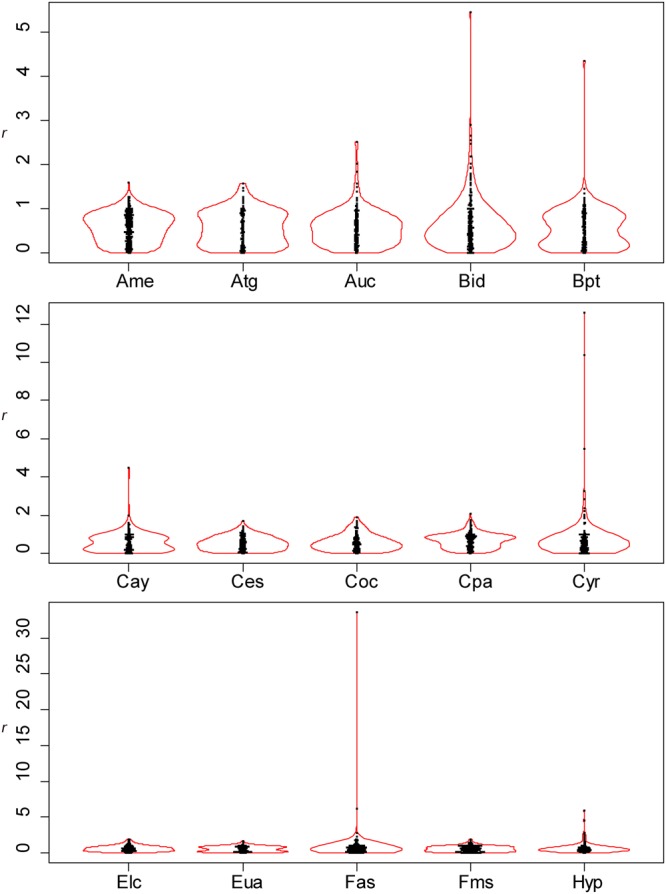
Distributions of the relative position of lateral veins (*r*) (see Fig. 2 for legend). All the values of *r* are shown. An outlier (extremely large value of *r*) was observed when two successive lateral veins on the same side of the leaf were very close to each other. Species are arranged in alphabetical order, and data for the first fifteen species (Ame–Hyp) are shown (see Fig. 5 for Jms–Udj).

**Figure 5:**
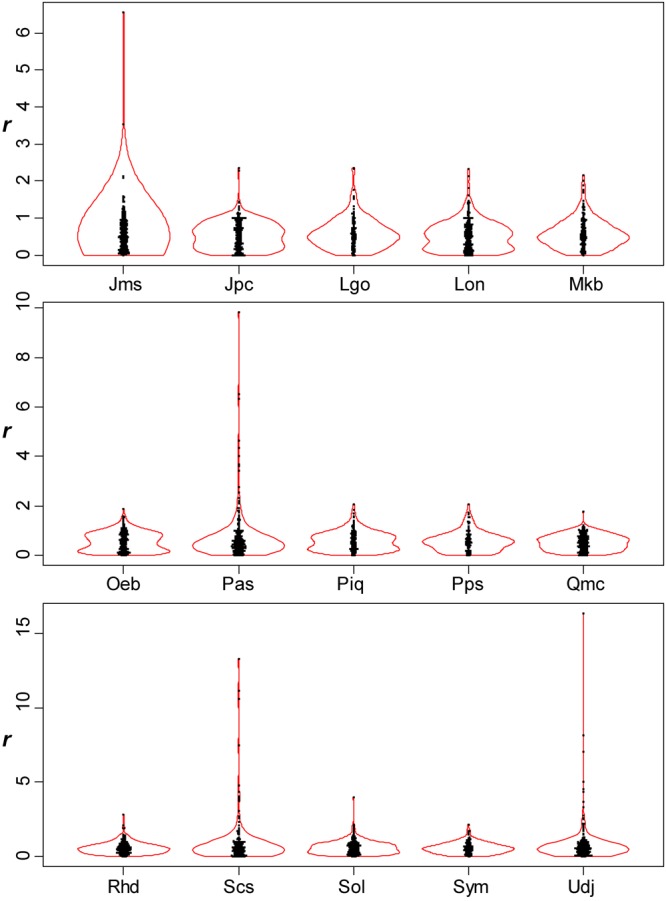
Distributions of the relative position of lateral veins (*r*) (see Fig. 2 for legend). All the values of *r* are shown. Data for the last fifteen species (Jms–Udj) are shown.

**Figure 6:**
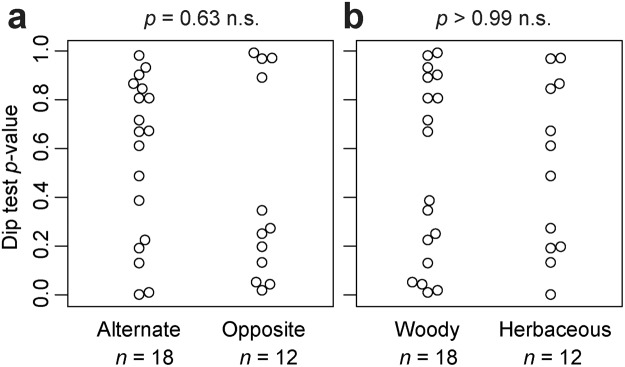
Comparison of the Hartigan’s dip test result *p*-values. The value of each species is shown as an open circle, each of which is arranged so as not to overlap others (i.e., bee swarm plots). (**a**) Species with alternate and opposite phyllotaxis and (**b**) woody and herbaceous life forms. The *p*-value of the Brunner–Munzel test is shown above each panel; no significant difference was found between groups for each panel.

**Figure 7:**
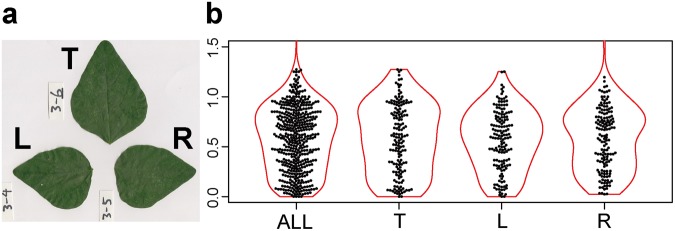
Comparison of the results obtained from analysis of the leaflets from different positions (T: terminal, L: left, R: right, and ALL: all positions pooled together) for *Amphicarpaea*, which had ternate leaves (an example of the scanned image is shown in the panel (a)). (Photographs by Kohei Koyama).

For all the species investigated, the arrangement of leaf secondary veins showed considerable intraspecific variation; the ratio (*r*) varied continuously from zero to more than 1 (Figs 2–5). For each of the species investigated, the arrangement of the secondary veins was therefore a mixture of symmetrically arranged (opposite-like) and asymmetrically arranged (alternate-like) pairs. The degree of unimodality differed among the species. Some species (Atg (*Acer*), Bpt (*Betula*), Cay (*Cayratia*), and Eua (*Euonymus*)) showed distinct deviation from unimodality (low *p* values) with peaks at *r* = 0–0.2 and at *r* = 0.8–1.0 (Fig. 2). The arrangements of the secondary veins of those species were therefore relatively close to opposite phyllotaxis, irrespective of their stem-level phyllotaxes (alternate: *Cayratia* and *Betula*, opposite: *Acer*, and *Euonymus*). In contrast, other species (Bid (*Bidens*), Lgo (*Ligustrum*), and Mkb (*Magnolia*)) showed little departure from unimodality (high *p*-values) and had an *r* distribution with a single mode at an *r* of ≈0.5 (Figs 2 and 3). The arrangements of the lateral veins of those species were therefore closer to alternate phyllotaxis, irrespective of their stem-level phyllotaxes (alternate: *Magnolia*, opposite: *Bidens* and *Ligustrum*). The remainder of the species had *r* distributions that were intermediate patterns between those two extreme cases. Lateral vein arrangements were related neither to stem-level leaf phyllotaxis (alternate vs. opposite) (Fig. 6a) nor to life form (woody vs. herbaceous) (Fig. 6b). For *Amphicarpaea*, which has ternately compound leaves, we separately analyzed only the terminal leaflets. We obtained results similar to those we obtained when we analyzed leaflets from all positions; mixtures of alternate-like (Fig. 1a), opposite-like (Fig. 1b), and irregular (Fig. 1c) patterns were observed in all cases (Fig. 7). The indication was that analyzing a leaflet as if it were equivalent to a leaf did not affect the results.

**Figure 8:**
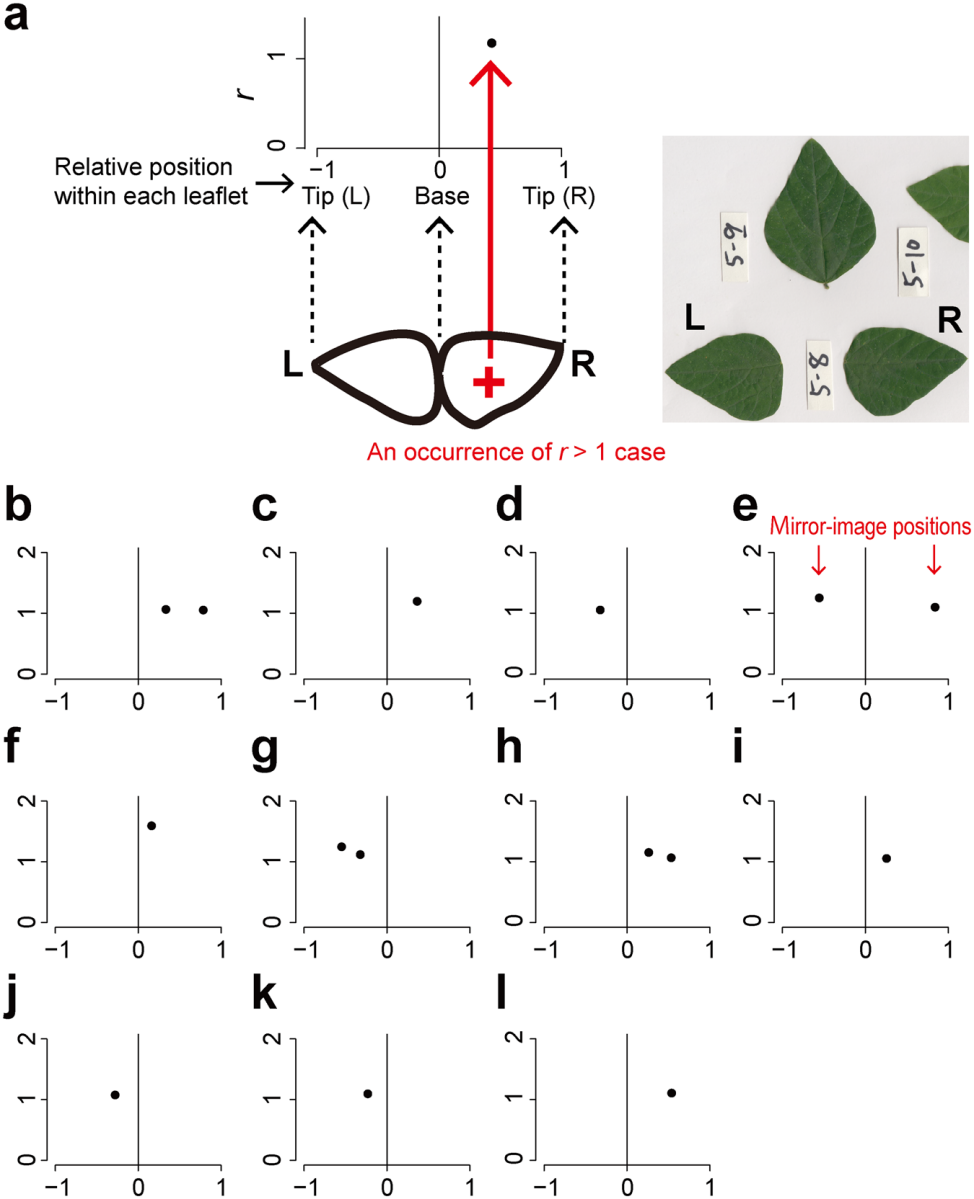
Comparison of left- and right-positioned leaflets for *Amphicarpaea*, which has ternate leaves. As illustrated in the panel (**a**), each closed circle indicates an occurrence of an irregular case (*r* > 1); the ordinate shows the value of *r* for each case, and the abscissa shows the normalized position within each pair of leaflets (−1: the tip of the left-positioned leaflet, 0: bases of the leaflets, and 1: the tip of the right-positioned leaflet). Each panel (b–l) shows the result of a comparison of the left- (L) and the right- (R) positioned leaflets of the same compound leaf. If those irregular cases had appeared as mirror images on each pair of leaflets, they should appear so on each panel. However, except for only one case (panel (e)), they did not appear as mirror images (panels (b–d) and (f–l)). The indication is that almost all cases were not generated by systematic rules. (Photographs by Kohei Koyama).

Irregular cases (*r* > 1), in which two or more successive lateral veins ran into the same side of the leaf lamina (Fig. 1c), were observed for all the species investigated (Figs 2 and 3). For *Amphicarpaea*, we found a total of 17 irregular cases on 12 leaflets among the 60 leaflets investigated. We investigated whether these irregular cases appeared on both the left- and right-positioned leaflets of the same compound leaf at approximately mirror-image positions. However, except for one case (Fig. 8, the panel [e]), the appearance of those irregular patterns on only one leaflet of the leaflet pair (Fig. 8) indicated that these patterns did not usually appear as mirror images.

## Discussion

None of the species investigated could be classified as having a strictly “alternate” or “opposite” lateral vein arrangement. Rather, as illustrated by the intraspecific variation of *r* (Figs 2 and 3), the actual arrangements of the lateral veins appeared to be mixtures of alternate-like (asymmetric) (Fig. 1a) and opposite-like (symmetric) (Fig. 1b) patterns. For some species, those patterns were closer to an alternate phyllotaxis (i.e., peaks at approximately *r* = 0.5) or opposite phyllotaxis (i.e., peaks at approximately *r* = 0 and 1), but those patterns did not always correspond with the stem-level phyllotaxis. Patterns corresponding to *r* > 1 (Fig. 1c) were rare but were observed for all species investigated (Figs 2 and 3). For pairs of right- and left-positioned leaflets of *Amphicarpaea*, those irregular patterns did not appear as regular mirror images; instead, they appeared as irregular or random events (Fig. 8).

These results are generally consistent with canalization models^41,42,60^. According to those models, locations of secondary veins are not strictly specified prior to leaf formation, and the leaf venation pattern is subject to considerable noise experienced during leaf development^40^. The canalization hypothesis has been further developed by Fujita and Mochizuki^43,44^, who conducted numerical simulations based on a model of the canalization hypothesis. The venations produced by their simulations were neither strictly alternate-like nor opposite-like patterns, but instead were a mixture of symmetric and asymmetric arrangements of lateral veins, a pattern that was observed in the present results. Furthermore, their simulations also produced the irregular pattern, in which two or more successive lateral veins ran into the same side of the leaf lamina (Fig. 1c). The canalization hypothesis could therefore explain the observed large variations in the positions of lateral veins.

Studies by Chitwood *et al*.^61^ and by Martinez *et al*.^49,62^ have further demonstrated that the asymmetric distribution of auxin in the leaf primordium systematically determines asymmetric placing of leaflets along the midrib of a compound leaf. Those studies have suggested that the position and the asymmetry of a leaf are determined prior to the development of each leaf. Results from those studies may imply that the arrangements of lateral veins along the midvein could also be determined systematically. We hypothesized that if the observed irregular cases (*r* > 1) had been generated by a systematic rule, they should have appeared as mirror images on each pair of left- and right-positioned leaflets. Nonetheless, we found that the irregular cases usually appeared on only one leaflet of each pair (Fig. 8). Hence, the irregular cases usually did not appear as mirror images, at least in the present dataset. The indication from these results that the irregular cases may have been extreme examples of noise supports the numerical simulations of the canalization model by Fujita and Mochizuki^43,44^.

In the canalization models^35,43–45^, the positions of lateral veins on the lamina are determined not only by the initial distribution of auxin sinks, but also by the interactions between auxin sinks and developing leaf lamina. These interactions are subject to considerable noise^40^. Our results showed that the arrangement of lateral veins along the midvein was a mixture of symmetric and asymmetric patterns. The indication seems to be that the mechanism underlying spacing of secondary veins around the midveins may differ from the mechanism underlying spacing of leaves, in agreement with previous studies^15,34,35,45^ (see Introduction). Actual venation patterns may be a consequence of both pre-determined spacing of midveins associated with asymmetry in auxin concentrations and post-determined spacing of auxin canals within each leaf lamina. Further studies are needed to clarify the extent to which auxin flow within a single leaf is determined prior to leaf development.

**Figure 9:**
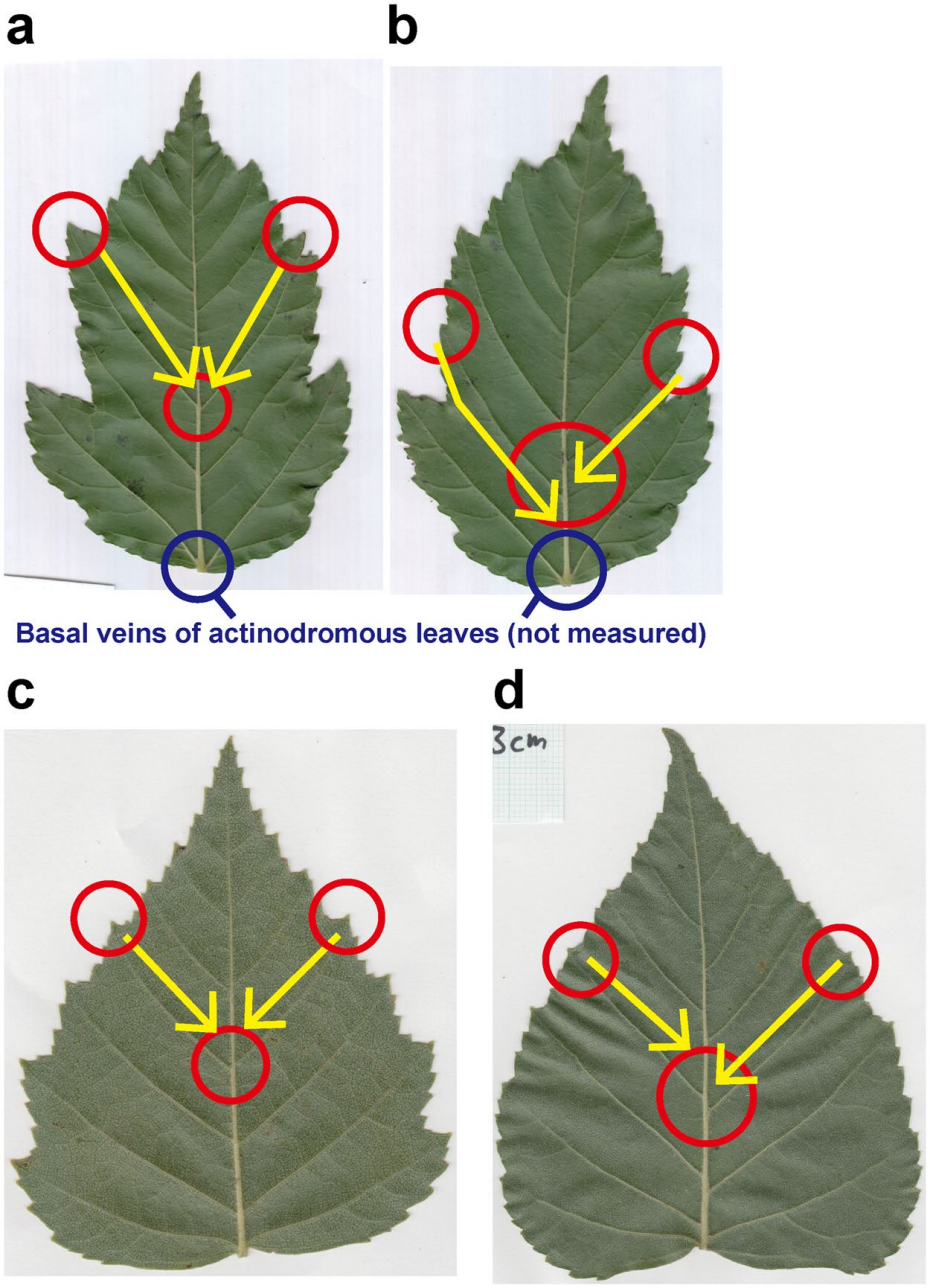
Lobation and lateral veins of Amur maple (*Acer*) (panels (a,b)) and Japanese white birch (*Betula*) (panels (c,d)). Several pairs of symmetrically arranged lateral veins often terminated at the tips of the lobes or serrations of the leaves (red circles in (**a**,**c**)). Note that even for the leaves of these species, asymmetrically arranged lateral veins (as shown in (**b**,**d**)) were also observed.

Correlated growth between lobes and major veins has long been recognized by developmental studies^15,63^ and is consistent with canalization models^40,43,48^. Those models have revealed that the locations of major veins are determined partially by the locations of lobes, the centers of which often become major veins. Those major veins follow a canal of auxin flow from the tip of the lobe toward the base of a leaf. The termination of some of the lateral veins of *Acer*, which has lobed leaves, at the lobe apexes is consistent with this scenario. When leaf laminae were symmetric, the symmetrically arranged lobes in some cases corresponded to symmetrically arranged lateral veins (Fig. 9a), though this was not true in every case (Fig. 9b). Similar patterns were observed for the serrated leaves of *Betula* (Fig. 9c,d). Therefore, although leaf shape may explain some aspects of the arrangement of lateral veins for some species, as predicted by theoretical studies, the present results indicate that leaf shape may not be the sole determinant of the arrangement of lateral veins.

The observed plasticity in the arrangement of lateral veins suggests that the arrangement of secondary veins *per se* may not significantly affect water or nutrient transport within leaf laminae as long as vein density is maintained. Despite the fact that both stem-level branching morphology and venation are constrained by optimization of resource transport^13,14^ and mechanical stability^64^, stem-level branching architecture is also constrained by three-dimensional light-capturing strategies^65^ and a balance between vertical and horizontal growth during competition with neighboring plants^66^. Inherent differences in functions may therefore partially explain the observed difference between venation and phyllotaxy. Nonetheless, because the present analysis was based on a limited dataset, further study is needed to confirm our conclusions.

## Data Availability

All the data, including the digital images (the scanned images and the images analyzed with ImageJ, which show all the measured points) are available from the Dryad Digital Repository: (data submitted to Dryad, provisional 10.5061/dryad.p6g8048) .
